# Does a simultaneous ventral/dorsal approach provide better reduction quality in treating acetabular fracture involving both columns with displaced posterior wall?

**DOI:** 10.1007/s00402-024-05224-6

**Published:** 2024-02-22

**Authors:** Yi-Hsun Yu, I-Jung Chen, Chih-Yang Lai, Yung-Heng Hsu, Ying-Chao Chou

**Affiliations:** Department of Orthopaedic Surgery, Musculoskeletal Research Center, Chang Gung Memorial Hospital, Chang Gung University and Medical College, Fu-Hsin St. Kweishan, Taoyuan, 33302 Taiwan

**Keywords:** Acetabular fracture, Pararectus, Gibson, Simultaneous, Reduction quality, Functional outcome

## Abstract

**Introduction:**

Various surgical techniques have been proposed to manage acetabular fractures involving both columns with posterior wall displacement. However, the optimal surgical approach to achieve satisfactory reduction quality remains controversial.

**Materials and methods:**

This retrospective study evaluated 34 patients with fractures who were treated at a single medical institution. The patients were divided into two groups according to the ventral/dorsal surgical approach employed: simultaneous (SI) and sequential (SE). Perioperative parameters, as well as radiological and functional outcomes, were analyzed and compared between the two groups.

**Results:**

The SI and SE groups comprised 9 and 23 out of the 34 patients, respectively. The SI group exhibited a significantly shorter surgical time and lower estimated blood loss than the SE group (*p* = 0.04 and 0.03, respectively). The quality of reductions of the anterior and posterior columns was similar between the two groups; however, superior reduction in the fracture gap of the posterior wall was observed in the SI group, as revealed by axial and coronal computed tomography scans.

**Conclusions:**

A simultaneous ventral and dorsal approach through the pararectus and the modified Gibson approach confer clinical advantages in reducing the fracture gap, surgical time, and intraoperative blood loss when managing acetabular fractures involving both columns and a displaced posterior wall. Therefore, these surgical approaches may be considered to be optimal for achieving satisfactory reduction quality in such fractures.

**Supplementary Information:**

The online version contains supplementary material available at 10.1007/s00402-024-05224-6.

## Introduction

Osteosynthesis for complex acetabular fractures continues to develop. Although several surgical approaches have been performed to treat acetabular fractures, treatment outcomes vary in the literature [1–4]. The ilioinguinal approach for fractures of the ventral part of the acetabulum was developed by Judet and Letournel and has become a classic surgical approach [5]. Following this approach, the anterior intrapelvic (AIP) approach in terms of the modified Stoppa approach was developed to avoid injury to the vital vessels and nerves with the second window of the ilioinguinal approach and became one of the most popular ventral approaches [6–9]. The pararectus approach, proposed by Keel et al., combined the advantages of the previously described approaches, and evidence of satisfactory outcomes from this approach has been reported in recent years [10–14].

Owing to advancements in the understanding of surgical anatomy, reduction instruments, and osteosynthesis implants, the fractured posterior column and the anterior column of the acetabulum can be reduced and fixed using the aforementioned ventral approaches [6, 8, 10, 11]. However, when these complex acetabular fractures involve the posterior wall, especially marginal impaction and displaced fragments, none of the mentioned ventral approaches can be applied to restore posterior wall congruency. Therefore, an additional dorsal approach is imperative to address posterior acetabular wall fractures. Since the Kocher–Langenbeck and modified Gibson approaches are applied to address dorsal acetabular fractures [15, 16], the dorsal approach is theoretically expected to maintain anatomical reduction and secure fixation; therefore, a better surgical outcome is anticipated.

Since dual approaches are essential to address displaced anterior column, posterior column, and posterior wall acetabular fractures, we were interested in investigating which sequence of approaches is most beneficial for patients. In this retrospective study, we proposed simultaneous treatment through the pararectus and modified Gibson approach to treat acetabular fractures involving the two columns and displaced posterior wall. The primary aim was to report the surgical outcomes of this cohort, and then secondarily to compare the perioperative, radiological, and functional outcomes with those of sequential ventral and dorsal approaches.

## Methods

**Fig. 1:**
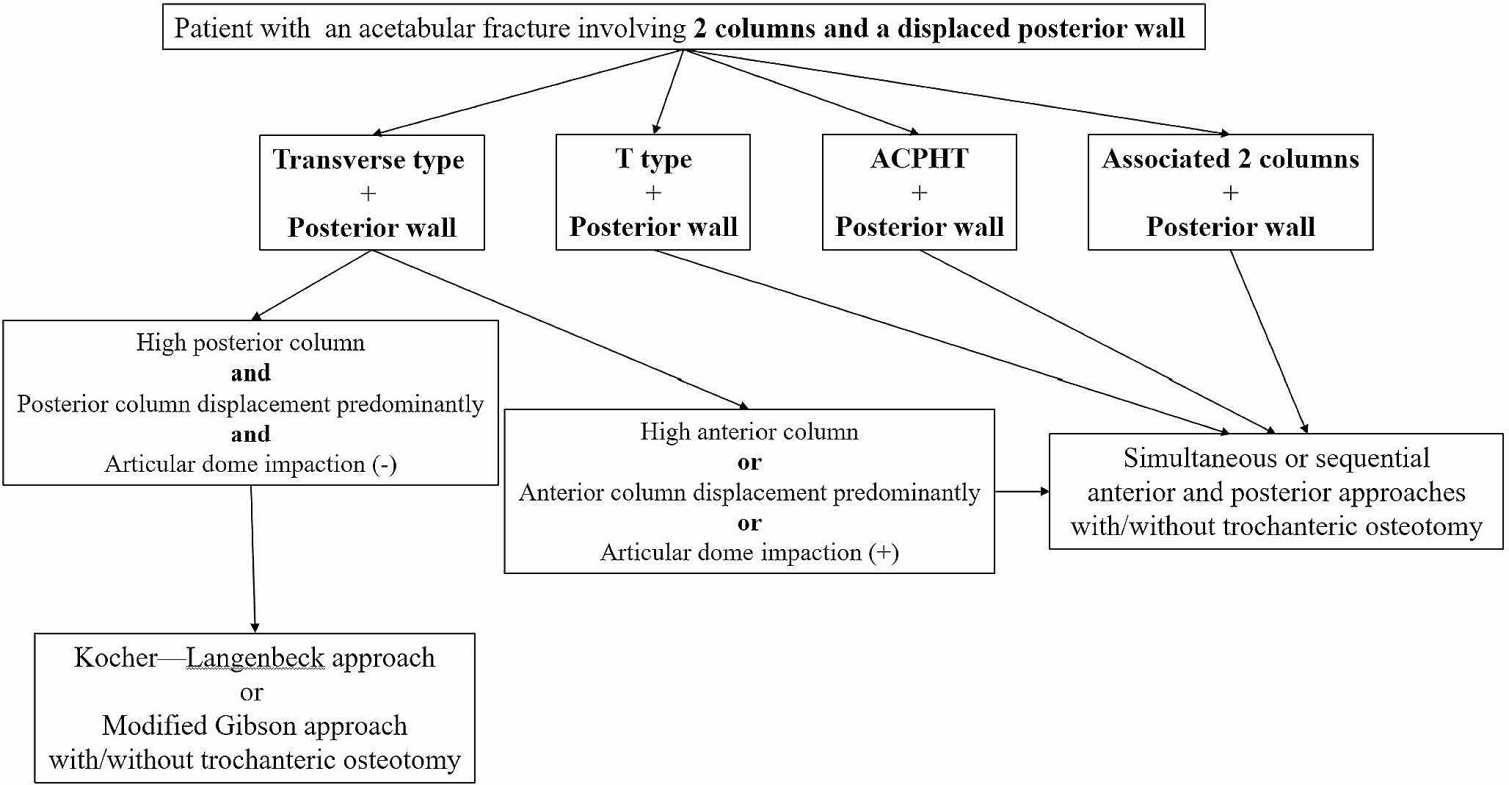
Flow chart for applying simultaneous or sequential ventral and dorsal approaches for the acetabular fractures involving both columns and a displaced posterior wall

The inclusion criteria for this study were patients with displaced acetabular fractures involving both columns (transverse type, T type, anterior column plus posterior hemitransverse type, and associated 2-column type) with a displaced posterior wall who underwent osteosynthesis at a single medical institution between 2018 and 2021. Specifically in the transverse type with a displaced posterior wall, a dorsal approach (the Kocher–Langenbeck or the modified Gibson approach) with the patient in the prone position is usually applied. However, a combination of ventral and dorsal approaches was applied for some specific injury patterns in this fracture type (Fig. 1). All the surgeries were performed by a head surgeon, who was assisted by 2 pelvic surgery fellows and 2 circulating orthopedic residents who participated in the surgical procedures. Clinical data were retrieved from the research database of the institution (Trauma Case Registration), and the review process was approved by the Institutional Review Board (approval no.: 202300326B0).

During the study period, 34 patients were identified and met the inclusion criteria. All patients were admitted to the emergency department. The resuscitation process essentially followed the guidelines of Adult Trauma Life Support. For patients with pelvic ring injury and acetabular fractures presenting with an unstable hemodynamic status and unresponsiveness to primary resuscitation (fluid and blood transfusion), emergent arterial embolization of the internal iliac artery(s) was performed as a hemostasis procedure. After stabilization, patients were transferred to the intensive care unit (ICU) for critical care or to a regular ward for preoperative preparation. Osteosynthesis was performed as soon as possible after the patients were stabilized.

Patients were divided into two groups: simultaneous ventral and dorsal (SI group) and sequential (SE group) ventral and dorsal approaches. Patient characteristics (sex, body mass index, preexisting comorbidities, Injury Severity Score [ISS], New Injury Severity Score [NISS], and associated injuries), perioperative parameters (approach, estimated blood loss, surgical time, and complications), and radiological and functional outcomes were recorded and analyzed.

### Perioperative image assessments and classification systems

All patients underwent comprehensive imaging assessment before and after osteosynthesis. Standard pelvic radiographs were obtained for acetabular fracture assessment, including the anteroposterior (AP), iliac, and obturator oblique views. Inlet and outlet views were acquired in patients with concomitant pelvic ring injuries. Multidirectional computed tomography (mCT) was performed for each patient to evaluate gaps among the fragments, step-offs of the articular surface, and the severity of dome/posterior wall/femoral head impaction injuries for preoperative planning. Follow-up image assessments were obtained using radiography (AP and two oblique views) at 3 and 6 months, at 1 year, and annually thereafter.

The Letournel classification was applied as the main system for acetabular fractures despite the fracture pattern involving both columns plus the posterior wall, which was not originally categorized in the 10-type classification system [17]. Additionally, pelvic ring injury was classified according to the Arbeitsgemeinschaft für Osteosynthesefragen (AO) classification [18].

We examined various parameters to assess the quality of acetabular fracture reduction. The Matta criteria were utilized based on the pelvic AP, iliac, and obturator views and were categorized as anatomical (gap = 0–2 mm), imperfect (gap = 2–3 mm), or poor (gap > 3 mm) [4]. In addition, the quality of fracture reduction was evaluated using mCT, including the axial, coronal, and sagittal planes, to determine the maximum residual gap and step-off after osteosynthesis.

### Rehabilitation protocol and functional outcome assessments

The rehabilitation program following osteosynthesis was individualized to meet each patient’s specific needs and accompanying injuries. Typically, patients were allowed to engage in toe-touch ambulation with the aid of crutches or a walker for four weeks after osteosynthesis. Gradual increases in weight-bearing during ambulation were subsequently introduced. The objective of gait training was to achieve unassisted ambulation within 12 weeks. Compression socks were used for mechanical prophylaxis for a minimum of 12 weeks; however, there is no standard medical prophylaxis for heterotopic ossification.

To evaluate functional outcomes, all patients underwent assessments using the Merle d’Aubigné [19] and Majeed [20] scores at 3, 6, and 12 months, and annually thereafter post-injury. The Merle d’Aubigné score assesses pain, mobility, and walking ability with scores ranging from 0 (worst condition) to 6 (best condition). Higher scores indicate better hip function. The Majeed score is a pelvic injury-specific functional assessment that evaluates seven items: pain, work, sitting, sexual intercourse, standing, unaided gait, and walking distance. The total score ranges from 0 to 100, with lower scores indicating greater disability.

### Operative procedure of simultaneous approaches

**Fig. 2:**
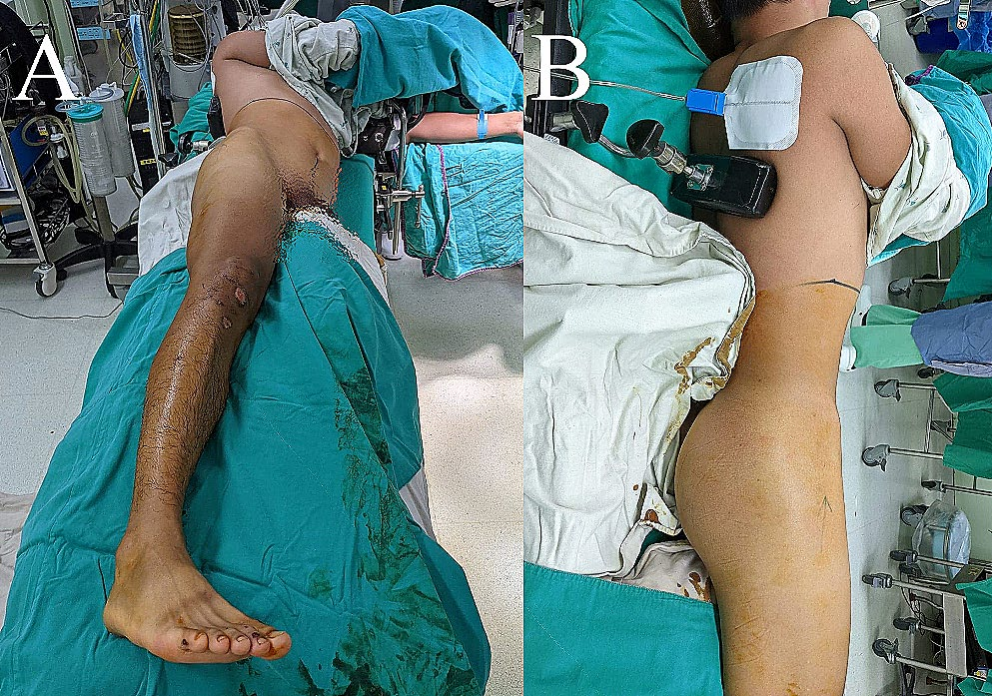
Patient position during osteosynthesis. **A** Bird’s eye view. **B** Posterior view

**Fig. 3:**
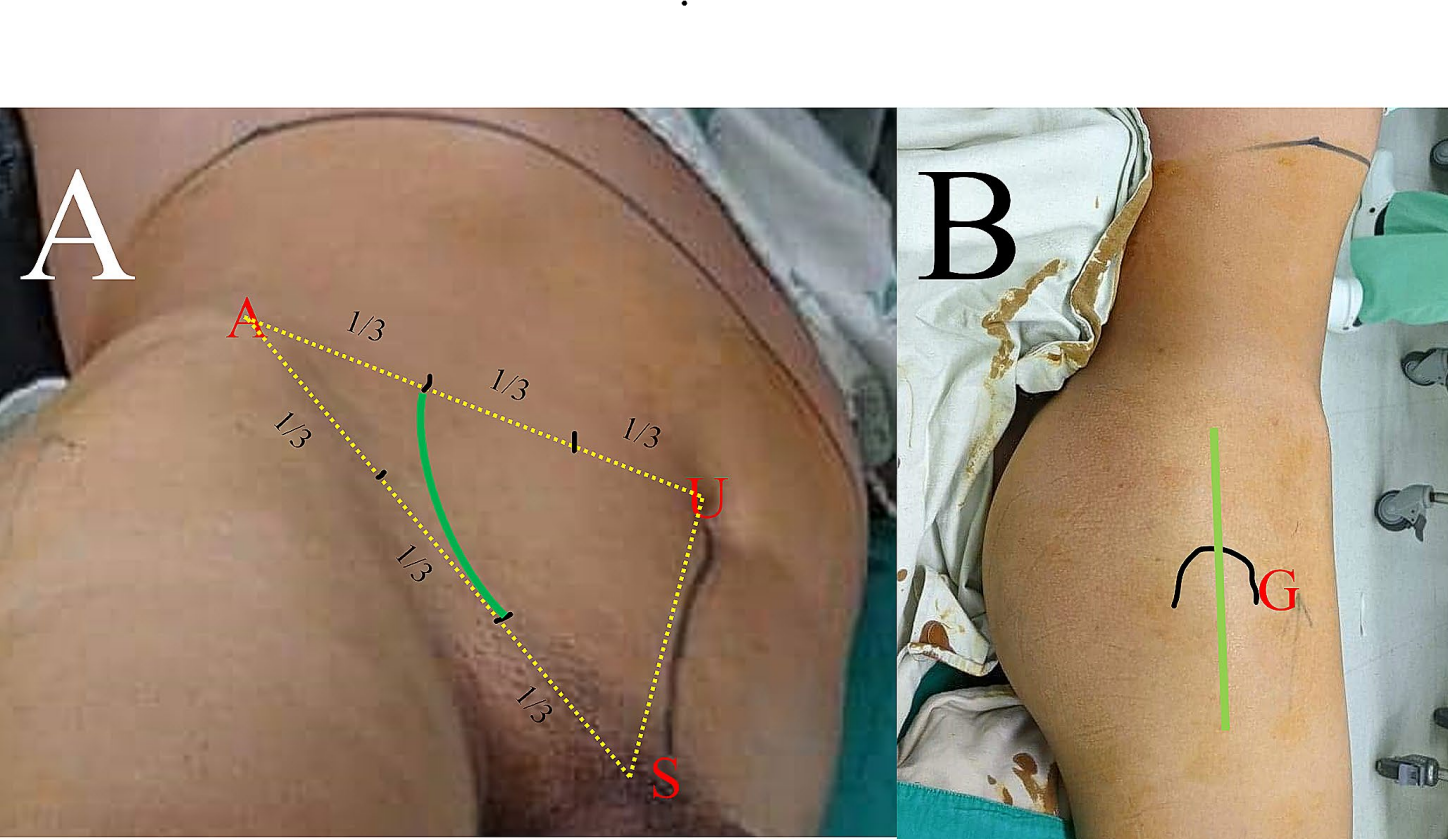
Landmarks of the pararectus **A** and modified Gibson **B** approaches using simultaneous ventral and dorsal approaches. A, anterior superior iliac spine; G, greater trochanter of the femur; S, pubic symphysis; U, umbilicus. Green line: surgical incisions

To expose both the ventral and dorsal pathologies of the acetabulum, the patient was placed on a radiolucent table in a semi-decubitus position with the injured hip facing upward (Fig. 2). Sterile drapes were prepared to expose the umbilicus, pubic symphysis, entire buttock, and injured leg. The landmarks for the pararectal approach were the umbilicus, anterior superior iliac crest, and pubic symphysis. The landmarks for the modified Gibson approach were the iliac crest and greater trochanter of the femur (Fig. 3).

Most often, the surgeon stood on the opposite side of the injured acetabulum, and the surgical incision was made ventrally (via the pararectus approach). The vital tissues (rectus abdominis, inferior epigastric vessels, spermatic cord [male] or round ligament [female], external iliac vessels, and iliacus) were identified sequentially to create working windows between them. A mini-incision above the iliac crest for manipulation of the fractured iliac crest or full surgical exposure of the iliac crest through the lateral window of the ilioinguinal approach may be applied for cases with very high anterior column fracture. All working windows were packed with gauze temporarily and firmly for hemostasis, and the surgeon shifted to the same site of the acetabular fracture to initiate the modified Gibson approach.

A straight incision along the alignment of the femur was made above the greater trochanter of the femur, 10 cm proximally and 10 cm distally. The Gibson interval was created between the tensor fascia lata and the gluteus maximus muscle fibers. The hip was then internally rotated to expose the short rotators. The pyriformis and obturator internus were transected with a scalpel (1.5 cm) from their femoral insertions, and sutures were created for subsequent repair during wound closure. A greater trochanteric osteotomy combined with surgical hip dislocation can be performed to remove intra-articular loose fragments, reduce and fix the femoral head fracture, or confirm the quality of the acetabular cartilage reduction.

After completion of the dorsal surgical approach, the sequence of reduction and fixation was initiated from the anterior column, followed by the posterior column, quadrilateral plate (if fractured), and finally the posterior wall. The anterior column was reduced using reduction maneuvers (Schantz pin traction, manual traction, and hip external rotation) and instruments (Weber clamp, Farabeuf clamp, Matta clamp, and ball-spike pusher [DePuy Synthes, Paoli, PA, USA]). After the anterior column was reduced, a neutralization plate (DePuy Synthes) was used to fix the anterior column. As the reduction and fixation maneuver for posterior column lesions through the Gibson approach via the semi-decubitus position would be difficult, the ventral approach is preferred for the major reduction and fixation procedure in posterior column fractures, and the reduction quality could be confirmed and adjusted slightly through the modified Gibson incision.

Reduction of the posterior column was performed through the working window between the iliacus and the external iliac vessels of the pararectus approach using a collinear-reduction clamp (DePuy Synthes) (to pull the dorsal displaced posterior column ventrally) and a ball-spike pusher (to push the medial displaced posterior column laterally). The quality of reduction in the posterior column was confirmed by finger palpation down to the greater sciatic notch using the modified Gibson approach. The posterior column was fixed with one or two posterior column screws and an infra-acetabular screw on the ventral plate.

**Fig. 4:**
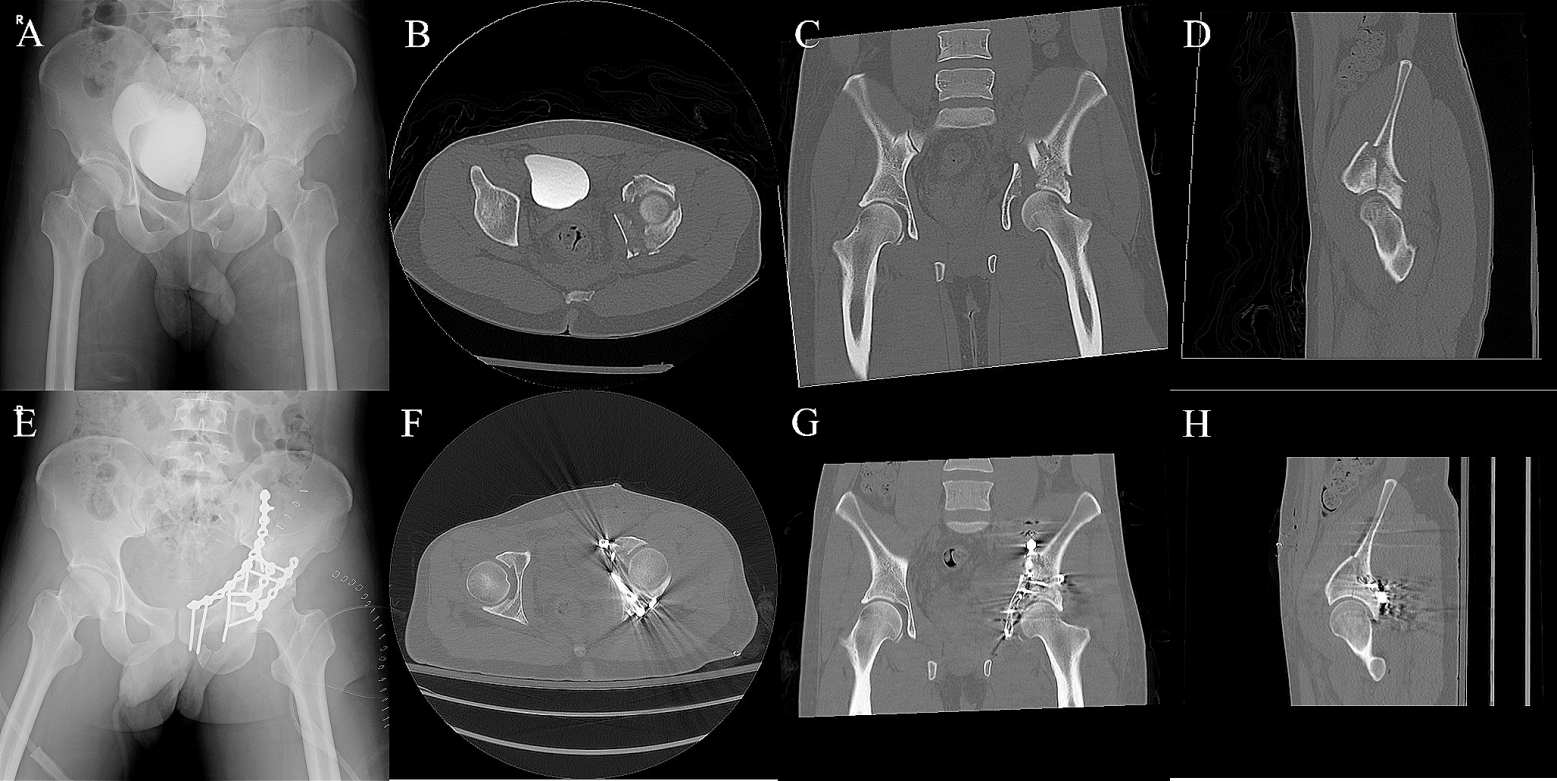
Images illustrating the use of simultaneous pararectus and modified Gibson approaches. **A** Anteroposterior pelvic X-ray pre-osteosynthesis; **B**–**D** axial, coronal, and sagittal views of the CT scan pre-osteosynthesis; **E** anteroposterior pelvic X-ray post-osteosynthesis; and **F**–**H** corresponding axial, coronal, and sagittal views of the CT scan post-osteosynthesis

Finally, osteosynthesis was performed on the posterior acetabular wall. The marginal impaction of the posterior wall was elevated with bone grafting before posterior wall reduction. The reduction of the posterior wall was basically performed using the Weber clamp and a ball-spike pusher. Reduction was confirmed, and two or three lag screws were positioned if the size of the posterior wall permitted. A neutralization plate (DePuy Synthes) was then positioned. Osteotomy of the greater trochanter was fixed (if performed), the short rotators were approximated and repaired, the transverse abdominis and external oblique muscles were repaired, and both wounds were closed layer-by-layer (Fig. 4). Surgical drains were not routinely used in either type of incision. However, for those who underwent surgery exceeding 3 h or with estimated blood loss > 1000 mL, surgical drains were placed to monitor residual bleeding and prevent hematoma formation.

### Statistical analysis

Data were analyzed using the SPSS software (version 23.0; IBM Corp., Armonk, NY, USA). Continuous variables were compared using the Mann–Whitney U test, and categorical variables were compared using the chi-square and Fisher’s exact tests. Statistical significance was set at *p* < 0.05.

## Results

The general characteristics of the enrolled patients are presented in Table 1. Regarding the referral medical center, 71.9% (23 patients) were transferred from nearby hospitals. The most common cause of fractures in this cohort was motor vehicle collision. Additionally, the most common accompanying injury was extremity fracture, followed by chest and head trauma. Table 2 shows a comparison of the chosen parameters between the SI and SE groups. Because simultaneous approaches were applied later than sequential approaches for such fractures, only the follow-up duration showed a difference.

**Table 1:** Characteristics of patients with acetabular fractures involving two columns and a displaced posterior wall

Patients, n	32
Age (years)	38.84 ± 16.74
Sex, *n* Male Female	24 8
Body mass index (kg/m^2)^	24.42 ± 5.31
Transfer Yes No	23 9
Injury mechanism Vehicle collision Fall injury Crush injury	29 3 1
Combined injuries Head trauma Face trauma Chest trauma Abdominal trauma Extremity fracture Spinal trauma	10 5 15 7 18 1

**Table 2:** Comparison of patient characteristics between the simultaneous ventral/dorsal (SI) and sequential ventral/dorsal (SE) groups

	SI group (*n* = 9)	SE group (*n* = 23)	p value
Age (years)	36.33 ± 7.63	39.83 ± 19.24	0.60
Sex, *n* Male Female	8 1	16 7	0.26
Medical comorbidity Yes No	2 6	6 17	0.95
BMI (kg/m^2)^	24.69 ± 3.84	24.31 ± 5.86	0.83
Transfer Yes No	8 1	15 8	0.18
Trauma mechanism			
Combined injury Head Face Chest Abdomen Extremity Spine	2 1 3 3 5 0	8 4 12 4 13 1	
ISS (median, IQR)	9 (27.5)	16 (14)	0.93
NISS (median, IQR)	18 (29)	22 (15)	0.85
Admission (days)	13.44 ± 6.41	17.96 ± 9.8	0.21
ICU Yes No	3 6	10 13	0.59
AE Yes No	1 8	3 20	0.88
Follow-up (months)	15.22 ± 5.87	29.09 ± 15.32	0.01*

*BMI* body mass index, *ISS* Injury Severity Score, *NISS* New Injury Severity Score, *ICU* intensive care unit, *AE* arterioembolization

*Statistical significance

Table 3 summarizes a perioperative comparison between the two groups. While most of the selected parameters were similar between the two groups, a significantly shorter surgical time and less estimated blood loss were observed in the SI group (*p* = 0.04 and 0.03, respectively).

**Table 3:** Comparison of perioperative parameters between the simultaneous ventral/dorsal (SI) and sequential ventral/dorsal (SE) groups

	SI group (*n* = 9)	SE group (*n* = 23)	p value
Fracture type ABC + PW ACPHT + PW Tr + PW T + PW	3 0 5 1	2 3 5 13	
Pelvic ring injury Yes No	3 6	11 12	0.46
Hip dislocation Yes No	7 2	16 7	0.64
Direction of hip dislocation Posterior Central	5 2	7 9	0.22
Impaction injury Acetabulum Both	3 6	11 12	0.46
Time to surgery (days)	5.89 ± 2.80	8.43 ± 4.44	0.12
Surgical time (min)	297.22 ± 82.64	371.39 ± 90.1	0.04*
Estimated blood loss (mL)	1122.22 ± 456.28	1484.7 ± 911.22	0.03*
Acute complication SSI Nerve injury	0 0	1 1	
Time to union	4.33 ± 1.32	2.17 ± 1.50	0.15
Osteonecrosis	3 (33.3%)	6 (26.1%)	0.68
Osteoarthritis	1 (11.1%)	5 (21.7%)	0.49

*ABC* associated both columns, *ACPHT* anterior column plus hemitransverse, *PW* posterior wall, *Tr* transverse

**Fig. 5:**
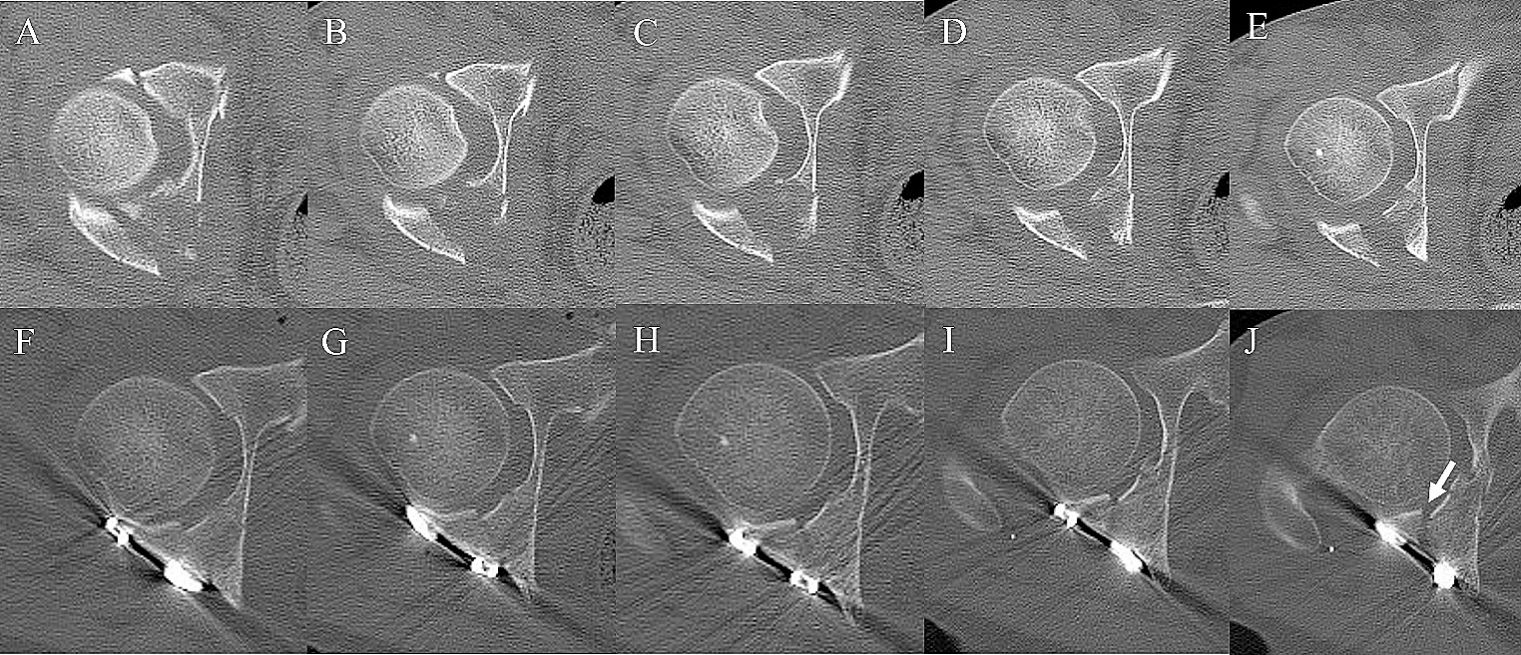
Illustrations of post-osteosynthesis reductions of the posterior wall of the acetabulum using sequential pararectus and modified Gibson approaches. **A**–**E** Series of axial views of the CT scan cranially to caudally showing a displaced posterior wall fracture with impaction injury. **F**–**J** Series of axial views of the CT scan cranially to caudally post-osteosynthesis. Arrow: residual fracture

The postoperative image assessments are shown in Tables 4 and 5. The reduction qualities from the radiographs (AP, iliac oblique, and obturator oblique) were interpreted using Matta’s criteria and both groups achieved similar reduction qualities. By using mCT to evaluate the improvement of the fracture gap and intra-articular step-off, a significant reduction in the intra-articular step-off in the axial view and fracture gap of the posterior wall in the coronal view could be achieved in the SI group (Fig. 5).

**Table 4:** Radiographical comparisons between the simultaneous ventral/dorsal (SI) and sequential ventral/dorsal (SE) groups

Matta criteria	SI group	SE group	p value
Anteroposterior Satisfactory Fair Poor	9 0 0	14 5 4	0.12
Iliac oblique Satisfactory Fair Poor	8 1 0	11 9 3	0.13
Obturator oblique Satisfactory Fair Poor	8 1 0	16 7 0	0.39

**Table 5:** Comparison of fracture gaps on postoperative multiplanar computed tomography between the simultaneous ventral/dorsal (SI) and sequential ventral/dorsal (SE) groups

	Axial			Coronal			Sagittal		
	SI group	SE group	*p* value	SI group	SE group	*p* value	SI group	SE group	*p* value
Maximal pre-osteosynthesis fracture gap (mm)									
Anterior column	13.33 ± 11.39	14.90 ± 8.50	0.67	14.11 ± 12.37	13.45 ± 6.91	0.85	12.30 ± 10.17	13.23 ± 9.21	0.80
Posterior column	8.33 ± 5.71	14.89 ± 8.73	0.047*	11.00 ± 8.74	16.83 ± 9.34	0.12	7.74 ± 6.39	11.70 ± 11.71	0.35
Posterior wall	17.93 ± 14.53	12.13 ± 9.10	0.19	15.21 ± 8.14	8.26 ± 8.29	0.04*	15.44 ± 13.34	10.11 ± 13.29	0.32
Articular step-off	13.3 ± 14.74	4.73 ± 3.58	0.01*	4.38 ± 3.24	4.94 ± 3.4	0.68	11.03 ± 12.13	5.32 ± 3.68	0.047*
Maximal post-osteosynthesis fracture gap (mm)									
Anterior column	1.87 ± 0.95	2.78 ± 2.05	0.21	1.1 ± 0.82	2.06 ± 2.51	0.27	0.8 ± 0.98	0.74 ± 1.19	0.89
Posterior column	0.83 ± 1.04	2.77 ± 2.62	0.04*	0.72 ± 0.77	2.91 ± 3.55	0.08	0.20 ± 0.56	0.26 ± 0.40	0.73
Posterior wall	1.21 ± 1.12	0.72 ± 1.63	0.42	0.87 ± 1.38	0.15 ± 0.23	0.03*	0.48 ± 0.72	0.15 ± 0.39	0.12
Articular step-off	0.71 ± 0.59	1.24 ± 1.33	0.27	0.81 ± 0.84	1.14 ± 1.51	0.55	0.64 ± 0.78	0.91 ± 2.05	0.71
Fracture gap reduction (mm)									
Anterior column	11.34 ± 11.02	12.51 ± 7.85	0.74	13.01 ± 12.64	16.62 ± 7.20	0.70	12.68 ± 10.11	13.14 ± 9.19	0.91
Posterior column	7.5 ± 5.51	11.86 ± 8.64	0.17	10.28 ± 8.51	14.59 ± 9.71	0.26	7.54 ± 6.47	13.61 ± 12.99	0.20
Posterior wall	15.31 ± 15.44	10.67 ± 9.62	0.33	14.03 ± 9.38	6.30 ± 6.39	0.02*	14.99 ± 14.42	8.21 ± 11.6	0.20
Articular step-off	11.91 ± 16.1	3.37 ± 3.24	0.02*	2.66 ± 2.96	3.97 ± 3.12	0.31	9.34 ± 13.29	4.46 ± 3.97	0.09

*Statistical significance

The functional outcomes evaluated using Merle d’Aubigné and Majeed hip scores are summarized in Table 6. Although the 3-month evaluations revealed better functional performance using simultaneous approaches, we did not observe further differences in the subsequent 6-, 12-, and 24-month evaluations.

**Table 6:** Functional evaluations and comparisons between the simultaneous ventral/dorsal (SI) and sequential ventral/dorsal (SE) groups

Follow-up period, months	Merle d’Aubigne score			Majeed hip score		
	SI group	SE group	*p* value	SI group	SE group	*p* value
**3**	8.13+-4.02	4.94+-2.25	0.02*	44.25+-14.52	33.59+-9.03	0.03*
**6**	12.25+-2.92	10.5+-4.41	0.32	65.38+-15.25	56.06+-17.13	0.20
**12**	14.13+-3.36	12.83+-3.11	0.35	76.38+-17.17	69.06+-16.97	0.33
**24**	16.00+-2.93	14.65+-2.67	0.26	83.63+-15.16	77+-15.52	0.33

## Discussion

The current study compared surgical results between simultaneous and sequential ventral/dorsal approaches for osteosynthesis of acetabular fractures involving two columns and a displaced posterior wall. We observed reduced surgical time and intraoperative blood loss in patients who underwent simultaneous ventral and dorsal approaches. Additionally, more ideal fracture reduction of displaced posterior wall fractures can be achieved using simultaneous approaches.

When fractures of the acetabulum involve the anterior and posterior columns, the necessity for a combined ventral and dorsal approach for reduction and fixation remains controversial. Some researchers have supported osteosynthesis using a single approach and obtained satisfactory outcomes [6, 8, 10, 11, 21]. However, some studies proposed that the combined ventral and dorsal approach provides greater advantages [22, 23]. A major surgical approach from the ventral side of the body is the primary surgical strategy for acetabular fracture involving two columns in our experience, and it is also a current surgical strategy for such fracture. Occasionally, an additional dorsal approach may be required for a significantly displaced posterior column or an unsatisfactory reduction of the posterior column from the ventral side. A well-planned patient position when executing the simultaneous ventral and dorsal approach could avoid predicaments such as the inability to restore the dorsal/ventral from the ventral/dorsal column, and the concern that the opposite column cannot be mobilized and reduced once the first column has been fixed. In particular, in the presence of a displaced posterior wall acetabular fracture, the posterior wall can be reduced and fixed securely, followed by well-reduced anterior and posterior columns, and favorable results can be obtained, which is challenging with a single ventral approach. Extensive single surgical approaches such as the triradiate approach or extended iliofemoral approach have been recommended by some surgeons for dealing with specific fracture patterns [24, 25]; however, massive soft tissue dissection may lead to complications such as heterotopic ossification, which decreases the functional recovery of patients.

Displaced acetabular fractures may result in end-stage osteoarthritis of the hip joint. Matta et al. reported 2- to 20-year follow-up outcomes after acetabular fractures [4]. In their series, the survival rate after posterior wall acetabular fracture was 76% (92% anatomical reduction), and 74–87% (67–70% anatomical reduction) for fractures involving two columns. To maintain a healthy joint after an acetabular fracture, anatomical restoration of articular congruence is critical. Using the same injury pattern, Shin et al. demonstrated their series and stated that posterior wall fragments could be neglected [26]. Similarly, Wang et al. applied a minimally invasive technique using lag screw fixation without surgical reduction [27]. In our series, the posterior wall fractures were all significantly displaced (least: 8.26 mm in the SE group in the coronal plane; largest: 17.93 mm in the SI group in the axial view). For displaced posterior wall fractures, neglecting treatment may lead to poor reduction and poor outcomes. Additionally, the impaction injury of the posterior wall fracture can be dis-impacted using minimally invasive osteosynthesis. Therefore, open reduction is highly recommended for displaced posterior walls with or without impaction injuries.

In addition, a displaced fracture of the posterior wall of the acetabulum can be effectively addressed using the dorsal approach while the patient is in a standard position such as decubitus or prone. However, our study showed that the posterior wall was anatomically reduced in the axial and coronal planes in the SI group. This was mainly due to the “butterfly effect,” in which the posterior column is difficult to mobilize after fixing the anterior column using sequential approaches, leading to compromised reduction. Although there were no significant between-group differences in the anterior and posterior column reductions, a larger gap in the posterior wall was likely to occur if residual gaps occurred in both the anterior and posterior columns. Meanwhile, longer surgical time and more intraoperative blood loss were observed in the sequential approach group. A longer surgical time undoubtedly resulted from the preparation of patients in different positions. Moreover, attempting to reduce the fracture gaps from the sequential approaches might also increase the surgical time. The increased blood loss in the SE group may result from the physiological responses associated with longer anesthesia and increased surgical time.

Clinical challenges may be encountered when using simultaneous approaches via the ventral and dorsal sites for acetabular fractures. First, sterile draping is compromised when the patient’s position changes. The operative time was extended because the main surgeon had to change his position. Finally, different surgical views were used to compare the routine position for each approach, such as the supine position for the ilioinguinal or AIP approach and the decubitus/prone position for the Kocher–Langenbeck approach. Therefore, we found that the use of the pararectus approach for ventral lesions and the modified Gibson approach for dorsal lesions offers some advantages. With respect to ventral approaches, the surgical view through the pararectus approach differs less in the semi-decubitus position than that through the ilioinguinal or AIP approach. We suggest that, if the surgeon is less familiar with the pararectus approach, the ilioinguinal approach is more suitable than the AIP approach because the surgeon’s view for fracture reduction is different. With the ilioinguinal approach, the majority of reduction maneuvers and reduction instrument placements are mainly within the middle window of this approach, which can be directly applied from the ventral to the dorsal side of the acetabulum. Nevertheless, the major surgical view of osteosynthesis using the AIP approach is from medial to lateral via the midline approach through either a longitudinal incision or a bikini incision. In the semi-decubitus position, the placement of implants may not be difficult; however, the surgical view during fracture reduction may be influenced or limited from the medial to lateral direction because the injured site is higher than the healthy site and the reduction may be blocked by the healthy site in the acetabulum, particularly in obese patients. We put forward that the pararectus approach may offer some advantages, especially when the advantages of the medial view during surgery (the AIP approach) and the workhorse for reduction maneuvers (the middle window of the ilioinguinal approach) are combined. Considering the dorsal approach, a straight incision jeopardizes the sterile surgical field less with the modified Gibson approach than with the curved Kocher–Langenbeck approach. Furthermore, greater trochanteric osteotomy (when surgical hip dislocation is indicated) is easier to perform than the Kocher–Langenbeck approach. However, we also found that having two surgeons familiar with these surgical approaches would be beneficial in shortening the surgical time and blood loss during the operation.

Despite efforts to avoid bias, this study has some limitations. First, it was a retrospective study with a relatively small number of patients. Simultaneous approaches have been applied at our institution since 2019. The quality of reductions, surgical time, and intraoperative blood loss may decrease with the accumulation of previous surgical experience. Finally, a longer follow-up period is required to determine the true incidence of post-traumatic arthritis after osteosynthesis by our proposed dual approach for complex acetabular fractures.

## Conclusion

Various surgical approaches can be applied for acetabular fractures involving two columns and a displaced posterior wall. Simultaneous ventral and dorsal approaches through the pararectus and the modified Gibson approach possess clinical advantages in reducing the fracture gap, surgical time, and intraoperative blood loss in treating such fractures.

### Electronic supplementary material

Below is the link to the electronic supplementary material.

Supplementary Material 1

## Data Availability

The datasets used and/or analyzed in the current study are available from the corresponding author upon reasonable request.
