# Supplementary material for: Does a simultaneous ventral/dorsal approach provide better reduction quality in treating acetabular fracture involving both columns with displaced posterior wall?
Source: Arch Orthop Trauma Surg. 2024 Feb 22;144(4):1547–56. doi: 10.1007/s00402-024-05224-6 (PMC10965567; doi:10.1007/s00402-024-05224-6)
Supplement: Supplementary file 1 — Supplementary Material 1 [file 402_2024_5224_MOESM1_ESM.docx]

**Conflicts of Interest Statement**

The authors certify that they have no commercial associations that may pose a conflict of interest in relation to the submitted article.
